# The distinctive gastric fluid proteome in gastric cancer reveals a multi-biomarker diagnostic profile

**DOI:** 10.1186/1755-8794-1-54

**Published:** 2008-10-25

**Authors:** Oi Lian Kon, Tai-Tung Yip, Meng Fatt Ho, Weng Hoong Chan, Wai Keong Wong, Soo Yong Tan, Wai Har Ng, Siok Yuen Kam, Alvin KH Eng, Patrick Ho, Rosa Viner, Hock Soo Ong, M Priyanthi Kumarasinghe

**Affiliations:** 1 Division of Medical Sciences, Humphrey Oei Institute of Cancer Research, National Cancer Centre, Singapore, Republic of Singapore; 2 Ciphergen Biosystems Inc., Fremont, USA; 3 Department of General Surgery, Singapore General Hospital, Singapore, Republic of Singapore; 4 Department of Pathology, Singapore General Hospital, Singapore, Republic of Singapore

## Abstract

**Background:**

Overall gastric cancer survival remains poor mainly because there are no reliable methods for identifying highly curable early stage disease. Multi-protein profiling of gastric fluids, obtained from the anatomic site of pathology, could reveal diagnostic proteomic fingerprints.

**Methods:**

Protein profiles were generated from gastric fluid samples of 19 gastric cancer and 36 benign gastritides patients undergoing elective, clinically-indicated gastroscopy using surface-enhanced laser desorption/ionization time-of-flight mass spectrometry on multiple ProteinChip arrays. Proteomic features were compared by significance analysis of microarray algorithm and two-way hierarchical clustering. A second blinded sample set (24 gastric cancers and 29 clinically benign gastritides) was used for validation.

**Results:**

By significance analysyis of microarray, 60 proteomic features were up-regulated and 46 were down-regulated in gastric cancer samples (*p *< 0.01). Multimarker clustering showed two distinctive proteomic profiles independent of age and ethnicity. Eighteen of 19 cancer samples clustered together (sensitivity 95%) while 27/36 of non-cancer samples clustered in a second group. Nine non-cancer samples that clustered with cancer samples included 5 pre-malignant lesions (1 adenomatous polyp and 4 intestinal metaplasia). Validation using a second sample set showed the sensitivity and specificity to be 88% and 93%, respectively. Positive predictive value of the combined data was 0.80. Selected peptide sequencing identified pepsinogen C and pepsin A activation peptide as significantly down-regulated and alpha-defensin as significantly up-regulated.

**Conclusion:**

This simple and reproducible multimarker proteomic assay could supplement clinical gastroscopic evaluation of symptomatic patients to enhance diagnostic accuracy for gastric cancer and pre-malignant lesions.

## Background

Unlike other common cancers, the prognosis for most gastric cancer patients is poor and has improved little over the past several decades. Five-year survival rates for gastric cancer are considerably lower than all major cancers except cancers of the liver, pancreas and esophagus [1]. Given that early stage gastric cancer has a much better prognosis (5-year survival approximately 90%) than advanced gastric cancer (5-year survival 3–10%) [2,3], global mortality from gastric cancer ought to decrease substantially by measures that result in downstaging of tumors at the time of initial diagnosis.

Although gastroscopy is the gold standard for gastric cancer diagnosis, its accuracy is not as high as it is for benign gastric diseases such as peptic ulcers, especially in geographic regions of low to intermediate gastric cancer prevalence. The percentage of missed cancer diagnosis, reported as 4.6%, 14% and even 33% [4-6], is not insignificant. Even in Japan, the false negative rate was reported to be 19% [7]. These data are consistent with the positive predictive value of only 0.4 – 0.7 for endoscopic diagnosis of gastric cancer in different centers [8-10]. Although the proportion of missed diagnoses appears small, the absolute number of patients denied the benefit of diagnosis at a curable stage is not negligible. Even at a modestly low false positive diagnostic rate of 5%, more than 47,000 gastric cancers would have been missed in one low prevalence country alone (USA) in a single year, 2000 [11]. Endoscopic assessment frequently includes mucosal biopsies but there are no clinical standards for either the optimal number of biopsies or the anatomic regions that should be sampled. A commonly cited recommendation is to take at least seven biopsies to correctly diagnose gastric cancer [12]. In this study however, fully 17% of all lesions subsequently shown to be malignant were considered benign on endoscopy. Thus, endoscopic mucosal examination suffers from inter-observer variation, suboptimal correlation with histopathology, difficulty in detecting submucosal cancers and unimpeded visualization of all anatomic sub-regions e.g. after previous gastric surgery [13,14].

Gastric fluid consists of a mixture of secreted soluble and exfoliated cellular proteins from the entire gastric mucosa – including regions that cannot be adequately assessed by fibreoptic gastroscopy. We therefore reasoned that the proteomic profile of gastric fluid, usually regarded as a waste by-product during gastroscopic examination, could usefully supplement conventional clinical evaluation by providing a 'molecular biopsy' that effectively samples the entire gastric mucosa, especially as protein detection techniques such as mass spectrometry can be highly sensitive. If performed during the course of clinically indicated gastroscopy, obtaining gastric fluid does not increase the invasiveness of the procedure. Unlike the plasma proteome, the gastric fluid proteome is likely to be less complex but enriched in disease-specific biomarkers, being generated directly at the disease site. The same biomarkers, even if present in plasma, may be diluted beyond the limits of detection and admixed with other more abundant systemic proteins that reflect concurrent pathophysiologic conditions (e.g. co-morbid diseases), rather than anatomic site-specific disease.

We have investigated a novel approach to developing biomarkers for gastric cancer by profiling soluble secreted peptides present in endoscopically aspirated gastric fluid and proteins extracted from exfoliated epithelial cells, also recovered during endoscopy by surface-enhanced laser desorption-ionization time-of-flight (SELDI TOF) mass spectrometry. Our results suggest that multiple protein biomarkers from an organ-specific source i.e. gastric fluid, generate a distinctive gastric cancer signature that merits further development as a tool for improving the diagnostic accuracy of gastroscopy and has potential for detecting early stage gastric cancer and pre-malignant lesions (intestinal metaplasia and dysplasia).

## Methods

### Clinical samples

Gastric fluids were obtained during gastroscopy of overnight fasted patients seen at the Singapore General Hospital. The study protocol was approved by the Ethics Committee of the Singapore General Hospital. and conformed to the provisions of the Declaration of Helsinki 1995. Indications for gastroscopy were solely clinical and were independent of the study. Initial analysis was performed on a training set of 19 samples from histologically proven gastric adenocarcinomas (13 intestinal type, 4 diffuse type, 1 mixed type, 1 indeterminate) [15] and 36 samples from patients with clinically benign gastric conditions. The mean age of 19 gastric cancer patients (13 male, 6 female; 17 Chinese, 2 Indian) was 68 years. Distribution by American Joint Committee on Cancer (AJCC) clinical staging was stage 0 (1 patient), stage I (4 patients), stage II (2 patients), stage III (2 patients) and stage IV (10 patients). The mean age of 36 patients with benign gastric conditions (19 male, 17 female; 33 Chinese, 2 Malay, 1 Indian) was 57 years. Clinical diagnoses after endoscopy of non-cancer patients were normal (9), antral gastritis (9), gastritis (6), ulcer (4), hiatal hernia (3), hyperplastic polyps (2), Barrett's esophagus (1), fundic scar (1) and adenomatous polyp (1).

The classification algorithm developed from the training set was tested by blinded analysis of a validation set consisting of another 24 histologically confirmed gastric adenocarcinomas (10 intestinal type, 7 diffuse type, 1 mixed type, 5 indeterminate, 1 neuroendocrine) and 29 clinically benign gastric samples. The mean age of these 24 gastric cancer patients (18 males, 6 females; 21 Chinese, 3 Malay) was 70 years. Distribution by AJCC clinical staging was stage I (5 patients), stage II (4 patients), stage III (2 patients) and stage IV (12 patients). One patient in the validation set declined further investigation and could not be staged. The mean age of 29 non-cancer patients (11 male, 18 female; 26 Chinese, 2 Indian, 1 Malay) was 47 years. Clinical diagnoses after gastroscopy of non-cancer patients were gastritis (14), fundic gland polyps (2), acute gastric ulcer (2), duodenitis (2), hiatal hernia (1) and normal (8).

None of the gastric cancer patients had received any form of cancer treatment at the time of gastroscopy.

Taking training and validation cases together, 19% (8/43) and 29% (19/65) of patients with gastric cancer and benign gastric conditions, respectively, were positive for *H. pylori*, a difference that was not significant by Fisher's exact test (2-sided *p *value = 0.4508).

### Sample collection and processing

Gastric fluid was aspirated into a sterile container at commencement of endoscopy, assigned an anonymised code and immediately placed on ice. Blood- or bile-stained samples were rejected. Only clinically suspicious mucosal lesions were biopsied at the discretion of the endoscopist. Gastric fluids were centrifuged at 180 g for 6 minutes at 4°C, from which the supernatant was centrifuged again at 16 100 g for 30 minutes at 4°C. Pellets from both centrifugations were combined. The high-speed supernatants were stored separately from the pellets at -80°C.

### Protein profiling

After thawing, 10 μl of each gastric fluid sample was applied to different chemical surfaces of ProteinChip arrays (Ciphergen Biosystems Inc, California, USA): (a) copper(II) Immobilized Metal Affinity Capture (IMAC3) in the presence of 100 μl of 1 mol/L urea, 1 g/L 3-[(3-cholamidopropyl)dimethylammonio]-1-propanesulfonate (CHAPS), 0.3 mol/L KCl, protease inhibitor cocktail (Roche Diagnostics, Mannheim, Germany), 50 mol/L TrisHCl, pH 7.5; (b) Weak Cation Exchange (WCX2 and CM10) in the presence of 100 μl of 50 mmol/L sodium acetate, 1 g/L octyl glucopyranoside, protease inhibitor cocktail, pH 5; (c) Strong Anion Exchange (SAX2) in the presence of 100 μl of 50 mmol/L TrisHCl, 1 g/L CHAPS, protease inhibitor cocktail, pH 8; and (d) Hydrophobic Interaction (H50) in the presence of 100 μl of 5 mL/L trifluoroacetic acid. After washing with 100 μl of the same respective buffers, sinapinic acid was added to facilitate desorption and ionization. The chips were analysed by SELDI-TOF-MS (PBSII, Ciphergen Biosystems Inc). Cancers and controls were intermingled and run concurrently on the same chip and on multiple chips to minimize chip-to-chip variation.

The gastric fluid pellets were resuspended in 25 μl of 6 mol/L guanidine thiocyanate, 5 g/L octyl glucopyranoside, 0.1 mol/L Hepes pH 7, and 100–200 μl of 9 mol/L urea, 2 g/L CHAPS, 50 mmol/L TrisHCl, pH 7.5 by vortexing for 45 minutes at 4°C. After centrifugation at 20 000 g for 5 minutes, 10 μl of the extract was applied to ProteinChip arrays as described above.

A retentate map was generated in which individual proteins were displayed as separate peaks on the basis of their mass to charge ratio. Data of the proteomic spectra were analyzed by Ciphergen Express Data Manager Software with Pattern Track and two-way hierarchical clustering algorithm. Aligned peaks with signal to noise ratios above 3 were normalized by total ion current. Proteomic features were further analyzed using the significance analysis of microarrays (SAM) software from Stanford University. The package was designed to address problems specific to microarray data analysis (signal to noise ratio variance different from gene to gene, large number of data points from a small number of samples) but we found it to be applicable to proteomic data analysis as well. The algorithm of the software was described by Tusher *et al*. [16]. In brief it defined a metric called the relative difference for measuring the difference between two or more groups of data in place of the *p *value. It employed a variation of the bootstrapping method and repeatedly divided a given data set (spectra containing the proteomic features in this study) randomly into two groups to calculate the relative difference for each of the permutations. The number of permutations was set to be 1000 in this study and the software computed 1000 relative difference values for each proteomic feature. The relative difference of the particular grouping of interest (observed relative difference) was compared to the average relative difference from all the permutations (expected relative difference) of each feature and the feature was judged to be up- or down-regulated according to whether its observed relative difference was greater or smaller than its expected relative difference by some threshold. The software estimated a false discovery rate (also defined in reference [16]) for each threshold value that provided an indirect means to set the cutoff. The markers identified by this method were statistically significant. The false discovery rate was set to be less than 0.05 in this study.

To validate the markers identified by SAM, a second batch of 53 blinded samples were added to the data set for hierarchical clustering using the Ciphergen Express Data Manager software. While the known samples used by SAM to select the markers were expected to perform well in the clustering, the blinded samples were included to test how well the markers generalize to unknown samples. The results of the clustering were simply compared against the true identity of the samples and no advanced classification method or any other software was used in the validation.

### Biomarker identification

Gastric fluid proteins were fractionated by anion exchange chromatography (Q HyperD, Ciphergen Biosystems Inc.), using stepwise changes in pH for elution. Proteins in the 50 mmol/L TrisHCl, 1 g/L octyl glucopyranoside, pH 8 eluants were further purified on a cation exchange array (LWCX30) using 50 mmol/L sodium acetate, 1 g/L octyl glucopyranoside, pH 5 as binding and washing buffer. After addition of alpha-cyano-4-hydroxycinnamic acid energy absorbing molecules (Ciphergen Biosystems Inc.), the retained proteins were analyzed by PBSII and Q-TOF (Waters/Micromass) equipped with a ProteinChip Interface (PCI 1000, Ciphergen Biosystems Inc). Proteins were characterized by MS/MS fragmentation and identification was done by database search with Mascot (Matrix Science Ltd., London, UK).

### Biomarker validation

This was performed in a third set of gastric fluid samples taken from benign gastric and gastric cancer patients. Each freshly collected sample was processed to remove solid debris and to concentrate the protein content as follows. After adding phenylmethanesulfonyl fluoride to a final concentration of 0.2 mM, the sample was centrifuged for 15 minutes at 500 g and 4°C. Protease inhibitors (Complete Mini™, Roche Applied Science, Indianapolis, IN, USA) were added to the supernatant followed by centrifugal membrane filtration at 2 900 g and 15°C (Amicon Ultra-4 centrifugal filter device, 5 000 nominal molecular weight limit; Millipore, Billerica, MA, USA) until the sample was reduced to 10 – 20% of its original volume. Total protein concentration was determined by the 2-D Quant Kit (Amersham Biosciences, Pisctaway, NJ, USA). Pepsinogen C and alpha-defensin 1–3 concentrations were determined by enzyme-linked immunoassay (ELISA) using kits from Alpco Diagnostics (Salem, NH, USA) and Hycult biotechnology b.v. (Uden, The Netherlands), respectively. Each processed sample was assayed in duplicate for pepsinogen C and defensin levels using the suppliers' protocols. Samples for pepsinogen C assay were pre-diluted 120-fold. Concentrations of pepsinogen C and alpha-defensin 1–3 were derived by reference to their respective standard curves and expressed as ng (pepsinogen C) or pg (defensin) per microgram of total gastric fluid protein.

### *Helicobacter pylori*

The presence of *H. pylori *in stomach tissues was identified by visualization of spiral microorganisms in histology sections and/or by immunohistochemistry. Four-micron tissue sections were de-waxed in xylene and decreasing grades of ethanol. Antigen retrieval was by heating in citrate buffer, pH 6.0. The primary antibody against *H. pylori *(1:50 dilution; DAKO A/S, Glostrup, Denmark) was followed by the secondary antibody polymer link (Envision Chem Mate, DAKO) and visualized using diaminobenzidine as chromogen.

## Results

**Figure 1:**
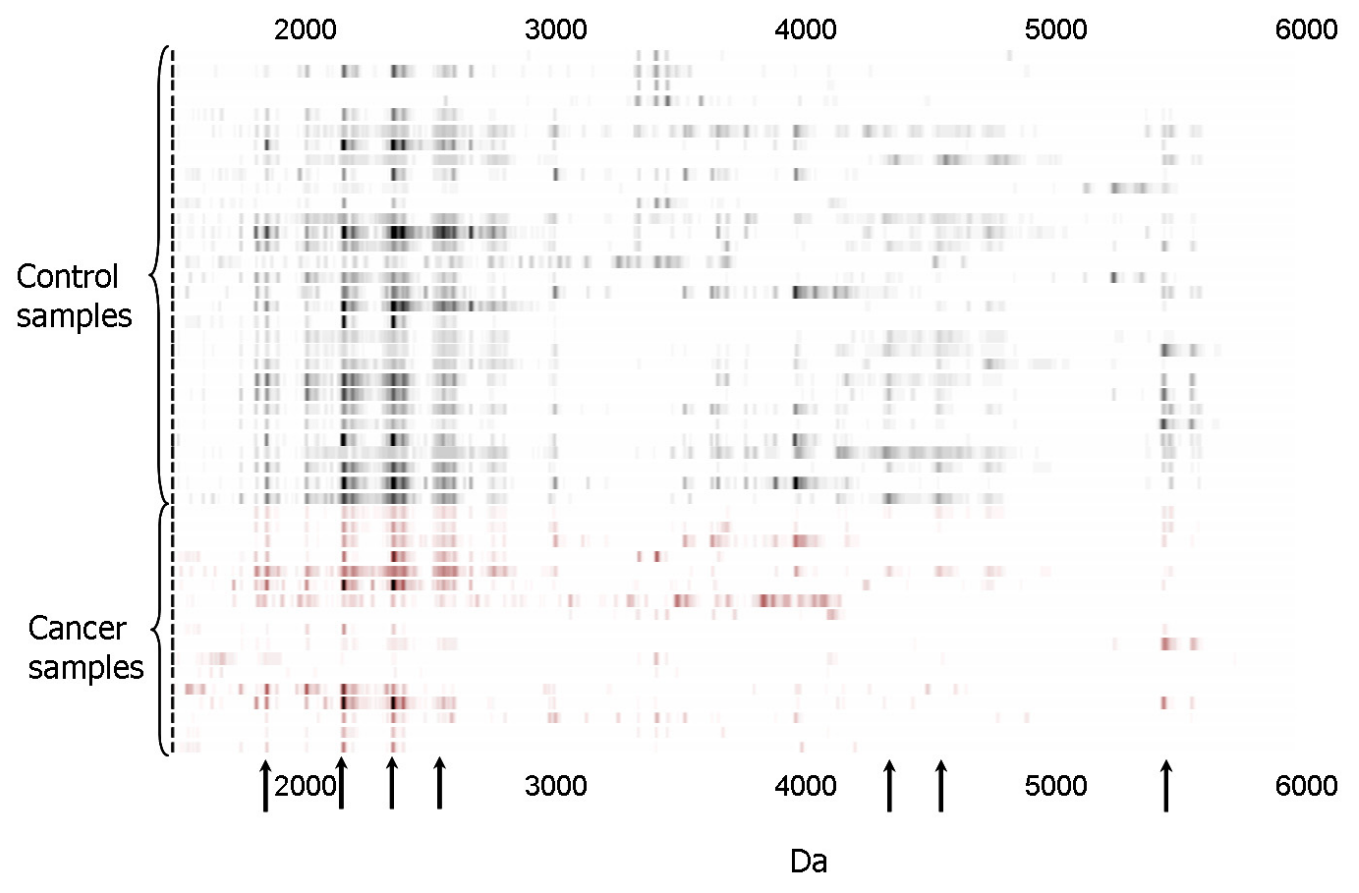
**Expression difference map of gastric fluid on copper(II) immobilized metal affinity capture ProteinChip array (IMAC3)**. Arrows indicate protein biomarkers significantly different in expression level between the two groups of samples.

Multiple up- or down-regulated protein biomarkers in gastric cancer were discovered in gastric fluid. A representative proteomic map of gastric fluid is shown in Figure 1. It is a gel view of a mass spectrum showing gastric fluid proteins selectively bound to immobilized copper(II) metal ion in the molecular weight range of 1500 Da to 6000 Da. Significant protein markers found to be down-regulated in cancer gastric fluid (*p *< 0.01) are indicated by arrows.

**Figure 2:**
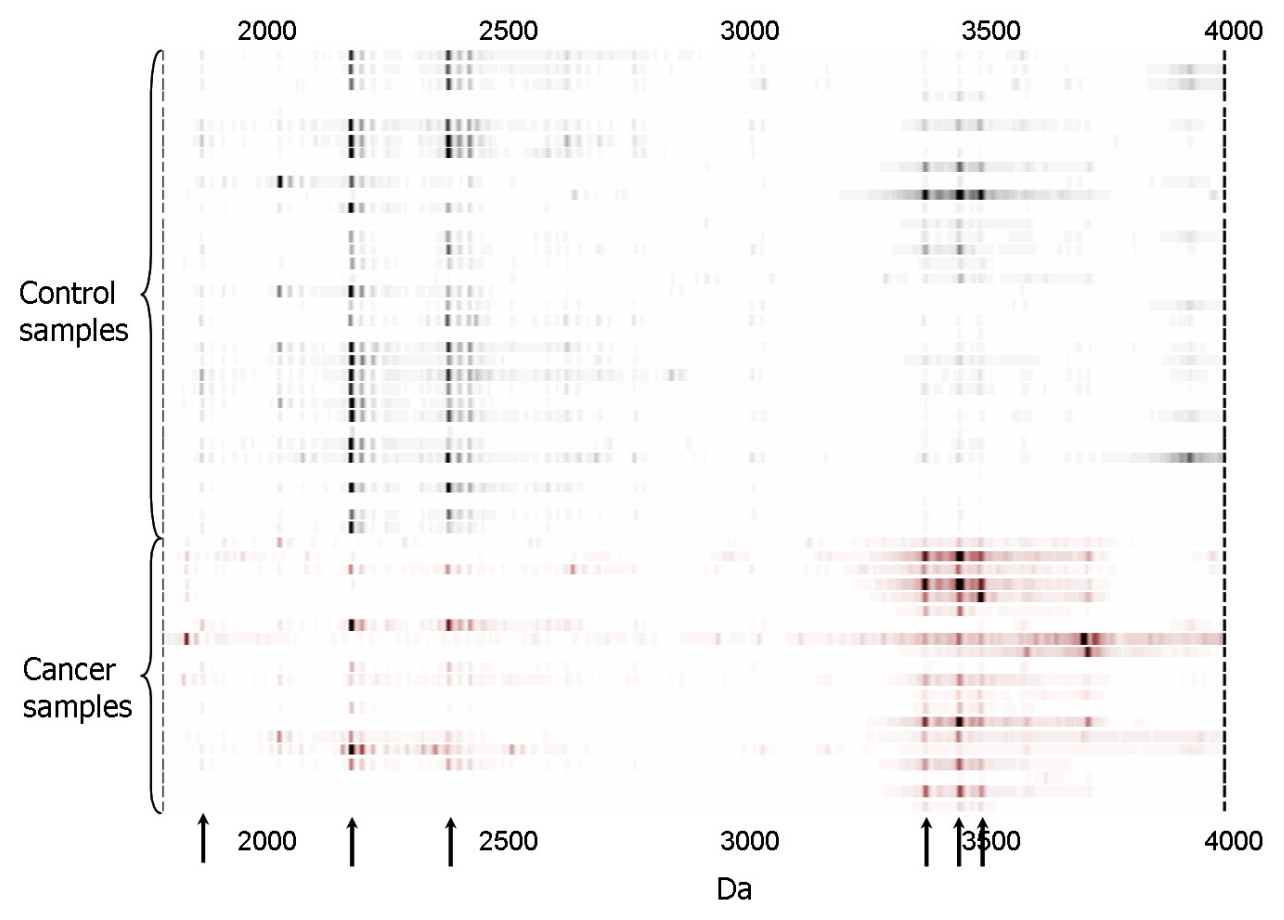
**Expression difference map of gastric fluid pellet extract on cation exchange ProteinChip array (WCX2)**. Arrows indicate protein biomarkers significantly different in expression level between the two groups of samples.

A representative proteomic map of gastric fluid pellet extract is shown in Figure 2. Proteins were selectively bound to a cation exchange array surface. Significant protein markers found to be up- or down-regulated in gastric cancer fluid pellet (*p *< 0.01) are indicated by arrows.

Average CV (coefficient of variation; cumulative for 10–15 major gastric fluid peaks per spectrum, n = 8) for immobilized copper(II) ProteinChip array (IMAC3) was 12.8%, for cation exchange array (WCX2) was 15%, for anion exchange array (SAX2) was 17.3%, and for hydrophobic interaction chip (H50) was 13.6%. These CV values are in line with reproducibility assessment in the SELDI literature [17,18].

**Figure 3:**
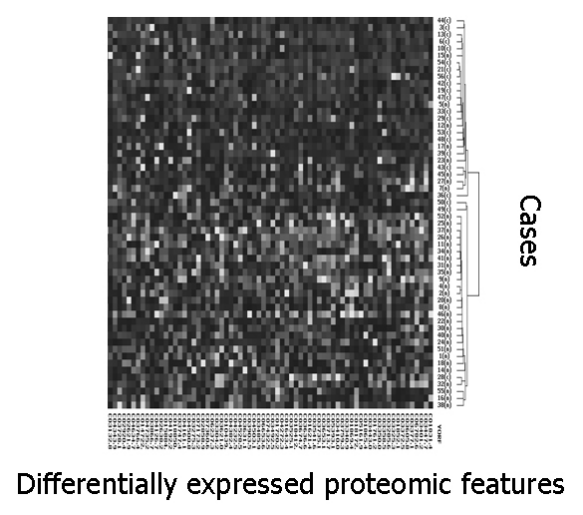
**Expression difference map of gastric fluid and pellet extract proteins of training set samples on four ProteinChip arrays, displayed in two-way hierarchical clustering**. Significant proteomic features are displayed vertically. The intensity of the grayscale indicates the degree of relative protein level, higher or lower than the median value. Patient cases are presented horizontally; most gastric cancer patients are tightly clustered together. This figure shows the upper quartile of the full image (please see Additional file 2 for the full image).

**Figure 4:**
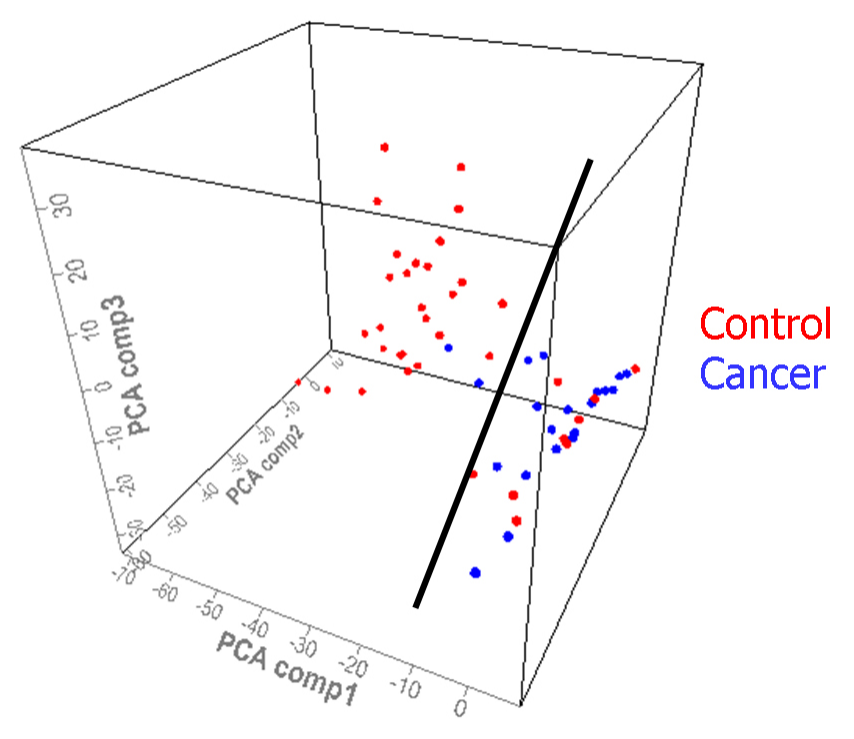
**Principal component analysis plot of proteomic features of training set samples**. A single plane (denoted by the black line) divides the samples into two groups with 1 false negative (shown in duplicate spots) and 9 false positives.

By SAM analysis of all proteomic features (total number of features 41 800, average number per retentate map 314) in gastric fluid and pellet extract, 46 proteomic features were found to be significantly down-regulated in gastric cancer and 60 proteomic features were significantly up-regulated in gastric cancer. (Data from different conditions, e.g., fluid and pellet as well as different surfaces, were simply aggregated together as distinct features for SAM. Markers reported by SAM in both pellet and supernatant fractions were manually identified and represented only once in the list after they were deemed biologically significant). Significantly down-regulated markers included 1884, 2428, 2594, 2840, 4050, 11720, 13700 Da; significantly up-regulated markers included 1761, 1831, 3372, 3443, 3605, 5160, 6780 Da. (Most of the significant markers were discovered on WCX2 and IMAC-copper(II), followed by SAX2). Based on the 106 significantly different proteomic features (Additional file 1), two-way hierarchical clustering analysis (two-dimensional complete linkage) was performed. Most of the gastric cancer cases were clustered together to form a distinctive group (Figure 3 and Additional file 2). Principal component analysis of the same data also revealed that cancer and benign samples could be well separated into two groups, with 2 false negatives (representing duplicate analysis of the same case) and 9 false positives, respectively (Figure 4). One gastric cancer fluid sample (from a case of stage I poorly differentiated gastric adenocarcinoma) clustered among non-cancer samples; all the other 4 early stage (stages 0 and I) patients correctly clustered with samples from 14 patients with stage II – IV gastric cancer, giving an overall diagnostic sensitivity of 95% (18/19 gastric cancer patients) on the training set.

Nine of 36 non-cancer samples in the training set clustered with the cancer samples (specificity 75%). Of these, 1 had a dysplastic adenomatous polyp – a precancerous lesion [19]. Among the other 8 patients, 6 had clinically directed biopsies that revealed intestinal metaplasia in 4 patients (67%). Eight non-cancer patients whose gastric fluid protein profiles clustered in the normal group also had clinically directed mucosal biopsies that showed intestinal metaplasia in only 2 patients (25%). A review of 1000 consecutive gastric biopsies performed for all indications showed an overall prevalence of intestinal metaplasia in the Singapore General Hospital during the study period of 30%. This contrasts with the prevalence of at least 67% of intestinal metaplasia among clinically benign cases whose proteomic profiles clustered more closely with gastric cancer cases than with other normals, consistent with intestinal metaplasia being an intermediate state in the transition of normal gastric epithelium to gastric adenocarcinoma. Accurate identification of intestinal metaplasia by endoscopy is known to be inaccurate [20]. Thus, a gastric cancer-type proteomic fingerprint is possibly a sensitive indicator for the presence of this pre-malignant lesion among patients clinically diagnosed as having benign gastric disorders.

Gastric cancer patients in the training set were significantly older (mean age 67.7 years) than patients with benign gastric conditions (mean age 56.6) (*p *= 0.0062). To address the possibility that protein profiles were related to age or ethnicity, we re-analyzed data of the subset of Chinese patients above 55 years of age. This resulted in 1/17 cancers misclassified (the same tumor that was misclassified when all 19 cancers were analyzed; sensitivity 94%) and 4/17 controls misclassified (the same 4 controls that were among the 9 misclassified benign cases; specificity 76.5%).

**Figure 5:**
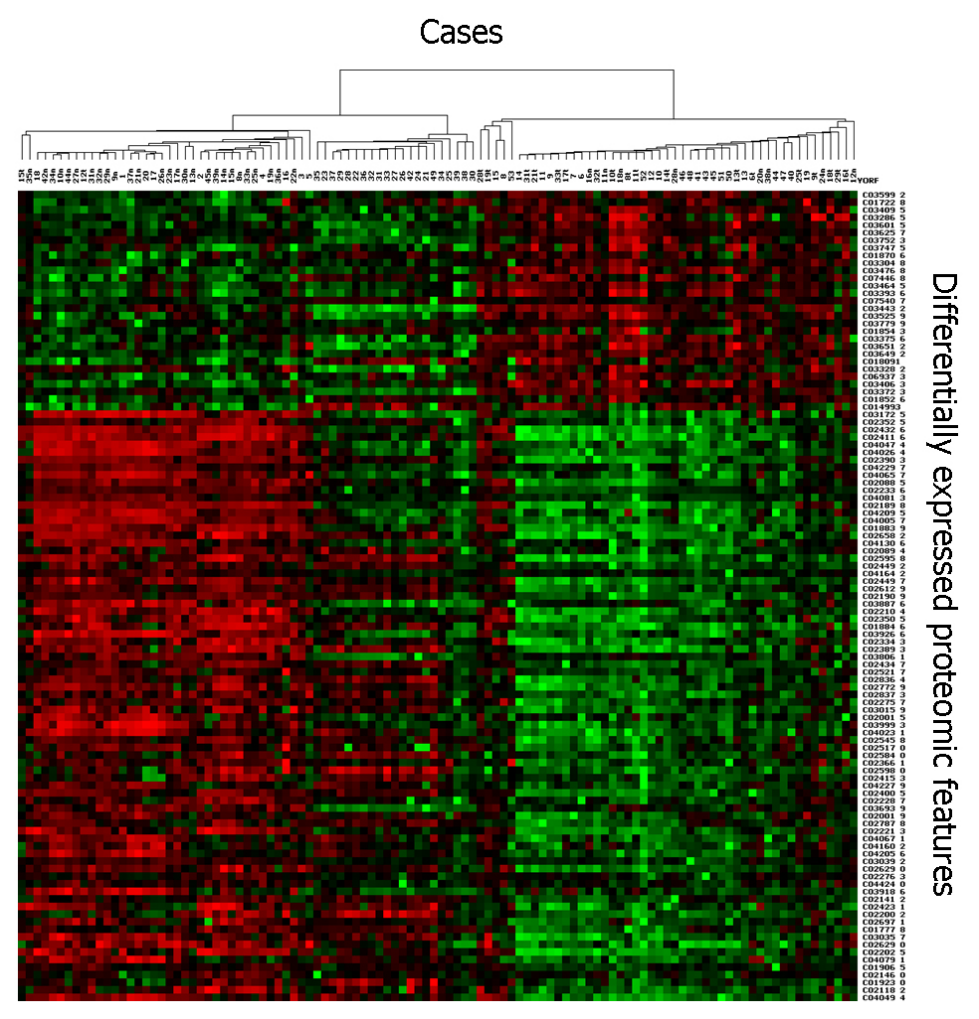
**Expression difference map of gastric fluid and pellet extract proteins of validation set samples on four ProteinChip arrays, displayed in two-way hierarchical clustering**. Significant proteomic features are displayed horizontally. The intensity of the red or green colours indicates the degree of relative protein level, higher or lower than the median value. Patient cases are presented vertically; most gastric cancer patients are tightly clustered together.

We next tested the actual performance of proteomic profiles in distinguishing cancer from benign samples in a second series of 53 blinded gastric fluid and pellet extract samples (24 gastric cancers and 29 benign gastric disorders) (Additional file 3). Twenty-one of 24 gastric cancers were correctly identified (sensitivity 88%) and 2 of 29 benign samples were wrongly classified (specificity 93%) (Figure 5).

**Figure 6:**
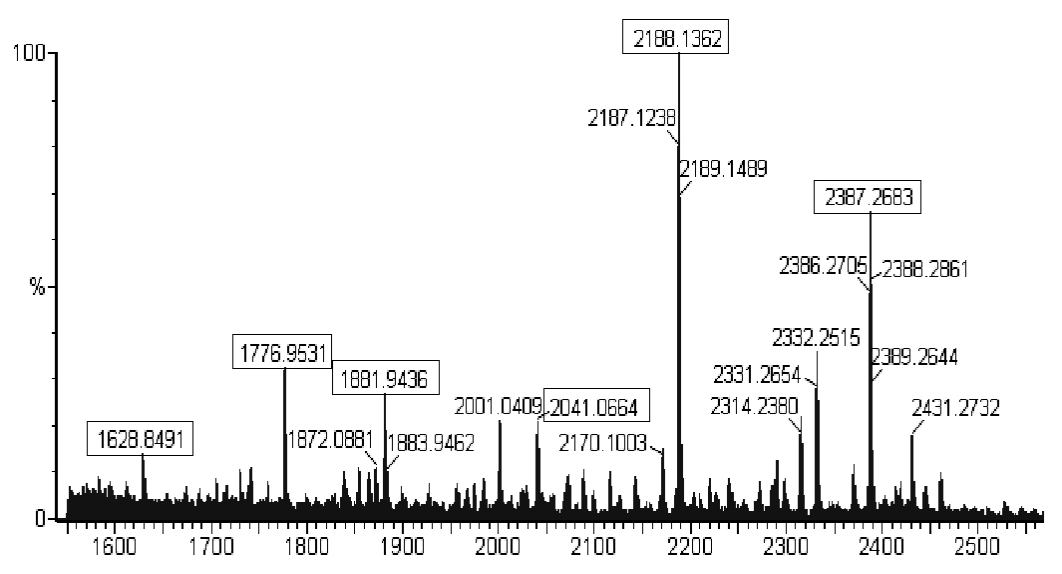
**High-resolution mass spectrum of fractionated gastric fluid proteins on LWCX30 ProteinChip array obtained on a QTOF equipped with a PCI1000 interface**. Boxed peaks were subjected to fragmentation analysis by collision-induced dissociation MS/MS.

**Figure 7:**
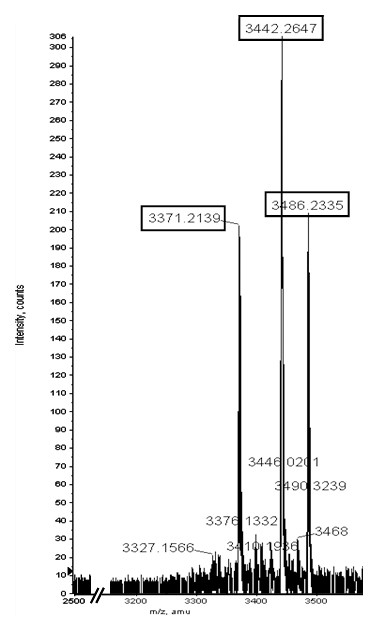
**High-resolution mass spectrum of gastric fluid proteins on H50 ProteinChip array obtained on a QTOF equipped with a PCI1000 interface**. This figure shows the up-regulated triplet markers in gastric cancer. Please see Additional file 4 for the full image.

**Figure 8:**
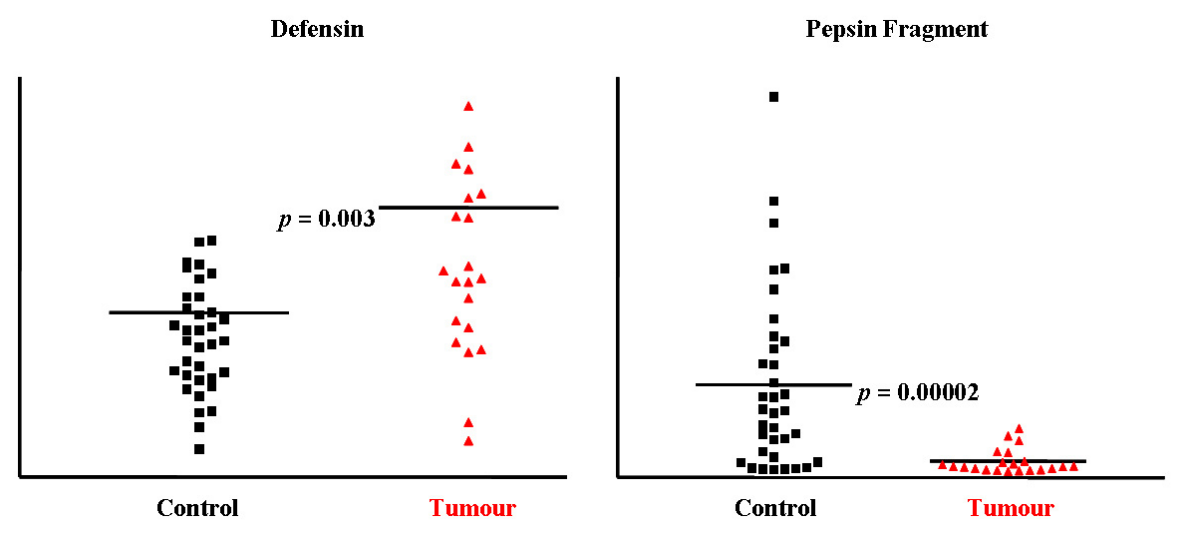
Scatter plots of intensity values of defensin and pepsin fragment present in gastric fluid samples of benign control and gastric cancer patients from the training set.

Selected proteomic markers (based on significance score determined by SAM) were semi-purified on ProteinChip arrays and identified directly on spots by collision-induced dissociation sequencing (Figure 6). Several of the significantly down-regulated markers in cancer patients shown in Figures 1 and 2, 1881.9 Da, 2041.0 Da, 2188.1 Da and 2387.3 Da, were identified to be pepsinogen C and pepsin A activation peptide fragments (Table 1). The up-regulated triplet markers in cancer patients shown in Figures 2, 7 and Additional file 4 were identified to be alpha defensin-1,2,3. Intensity scatter plots show highly significant differences in the mean intensities of defensin and pepsin fragment between benign control and gastric cancer fluid samples (*p *= 0.003 and 0.00002, respectively) (Figure 8). Using ELISA specific for pepsinogen C, we confirmed significantly lower concentrations in gastric cancer fluids (11.9 ± 0.1 ng/μg total protein; mean ± s.e.m. n = 6.) compared to benign samples (21.5 ± 1.4 ng/μg total protein. n = 23) in a third sample set (*p *= 0.0126; Student's unpaired two-tailed *t *test). ELISA performed on the same sample set for defensin levels showed higher concentrations in gastric cancer samples (63.4 ± 9.2 pg/μg total protein; mean ± s.e.m. n = 6) than in benign samples (46.2 pg/μg total protein; mean ± s.e.m. n = 23) ((*p *= 0.0654; Student's *t *test).

**Table 1:** Peptide sequences identified by MS//MS

**Peptide *m/z***	**Sequence**	**Protein Match**	**Mowse^† ^score**	**Mowse score with significant homology**
2386.29	FLKKHNLNPARKYFPQWKA	Pepsin A activation peptide	35	>28
2187.12	FLKKHNLNPARKYFPQW	Pepsin A activation peptide	18	>26
2040.03	LKKHNLNPARKYFPQW	Pepsin A activation peptide	28	>26
1775.95	FLKKHNLNPARKYF	Pepsin A activation peptide	47	>26
1628.84	LKKHNLNPARKYF	Pepsin A activation peptide	40	>28

1880.92	LRTHKYDPAWKYRF	Pepsinogen C activation peptide	31	> 22

^†^Mowse score = -10Log(P), where P = probability that the match is a random event (*P *< 0.05) *m/z*, mass/charge

## Discussion

Our data suggest that the spectral profile of unfractionated gastric fluid could be a useful adjunct for cancer diagnosis and detection of early stage disease, when combined with clinical gastroscopy. Most recent attempts at identifying protein biomarkers for gastric cancer have investigated serum [21-29] and tissue [24,30-37], and have increasingly used mass spectrometry. Older reports of serological assays of individual known tumor markers e.g. CEA, CA 19-9, CA 72-4, CA242 and TAG-72, have generally low sensitivity (<50%) [38-41]. Moreover, there is substantial cross-positivity of these tumor markers in non-gastric cancers e.g. raised CEA and MG7-Ag levels are common in colorectal cancer, cholangiocarcinoma, pancreatic carcinoma, and even in healthy controls [40,23]. Not surprisingly, such serum tumor markers have no established role in gastric cancer diagnosis and screening, although they may serve as prognostic indicators and early markers of recurrent disease following gastrectomy [39,41,42].

We chose to examine proteomic profiles of gastric fluid for disease biomarkers because it seemed likely that perturbed gastric protein secretion in malignant and pre-malignant states, coupled with the possible presence of exfoliated cancer cells, could generate distinctive proteomic profiles. As in the search for serum biomarkers, several groups have investigated the diagnostic utility of known tumor markers in gastric juice. Neither CEA nor CA 19-9 positivity in gastric fluid has demonstrated diagnostic accuracy [43-46]. Alpha-1 antitrypsin in gastric juice has recently been reported as a gastric cancer biomarker [47,48].

Our approach to developing a sensitive method for gastric cancer diagnosis differed from previous studies in three ways. First, we chose a biological sample that was organ-specific (i.e. endoscopically aspirated gastric fluid) rather than systemic (i.e. serum), reasoning that the molecular features would more likely be disease-specific. Second, mass spectrometry enabled us to take an unbiased discovery-based approach. Third, our data generated profiles of multiple proteomic markers that are increasingly regarded as having higher sensitivity and specificity than single tumor markers [49,50]. For gastric cancer, combining even 2 or 3 tumor markers achieved better diagnostic accuracy compared to a single marker alone [38,40].

Protein fingerprints of gastric fluid from gastric cancer patients showed a total of 106 proteomic features that were significantly up- or down-regulated (Additional files 1 and 3). Two prominent markers were selected for identification by MS/MS. Pepsinogen A and pepsinogen C activation peptides were down-regulated in gastric fluids removed from stomachs with histologically confirmed adenocarcinomas. A study of cryostat sections of gastric cancer has also reported significant down-regulation of pepsinogen C, identified by MS/MS, in tumor tissue [51]. Reduced pepsinogen levels in blood and tissue are a well-known consequence of multifocal chronic atrophic gastritis, a histopathological condition which increases the risk of developing intestinal-type gastric cancer [52]. However, chronic atrophic gastritis itself is not gastric cancer. A prospective 10-year longitudinal study determined that <3% of patients with this lesion progress to gastric cancer [53], while in another prospective series, 49% of severe chronic atrophic gastritis actually regressed to less advanced lesions when followed over 5 years [54]. Hence, although serum pepsinogen levels have been intensively investigated as a cost-effective serological biopsy for non-invasive gastric cancer screening, it has low positive predictive value – (0.77%–1.25%) in general population studies and 15% in selected patients [55].

We identified a prominent up-regulated marker as alpha-defensin 1–3. Immunohistochemical staining of gastric adenocarcinoma tissues showed that alpha-defensin expression was restricted to infiltrating neutrophils, and was absent in normal and malignant gastric epithelial cells (data not shown). Alpha-defensin overexpression from intra-tumoral neutrophils has also been reported in oral carcinomas by peptide sequencing and immunohistochemistry [56,57]. Association of defensin expression with cancers is consistent with the role of chronic inflammation in oncogenesis. Expression of alpha-defensins 1–3 was higher in colorectal cancer than in normal colon [58,59], and correlated with tumor invasiveness in bladder cancer [60]. Using SELDI TOF mass spectrometry, alpha-defensins 1 and 2 were among five prominent proteins in urine samples of patients with transitional cell bladder cancer [61]. Neutrophil defensin has also been identified in the proteomic signatures of ovarian and breast cancers [62,63].

Our data show that protein fingerprinting of gastric fluid achieves high sensitivity and specificity because it does not rely on a single marker. Multivariate analysis of a multimarker panel of both gastric fluid and exfoliated cellular proteomes has revealed a composite pattern of up- and down-regulation of multiple components to generate a highly specific and sensitive diagnostic method for gastric cancer, including early stage disease. The protein profiles in this study were independent of age and ethnicity, and performed well in correctly identifying whether gastric fluid was obtained from a malignant or benign source (sensitivity 88%; specificity 93%) when tested independently against blinded samples. Combining data from training and validation sample sets, the gastric cancer proteomic signature had a positive predictive value of 0.80. Most previous biomarker discovery studies have focused on a limited number of markers [21,23-26,38-41] and few have approached the diagnostic accuracy of this study. Relying on a single biomarker has several disadvantages. Individual markers are, in general, not very powerful in classifying disease from healthy controls. A biofluid study of bladder cancer markers using SELDI TOF mass spectrometry found that detection rates with single markers were significantly improved by biomarker combinations and clusters [61]. Our analysis confirms this experience. Thus, although the mean albumin level in gastric cancer fluid was significantly different from controls (*p *= 0.005), only 8 out of 19 cancer patients in the training set showed such elevation (data not shown), whereas transferrin and alpha1-antitrypsin levels were upregulated in an even smaller number of patients and were not significantly different from the general patient population (*p *> 0.1) (data not shown). Furthermore, many cancers share the same markers e.g. CEA, CA19-9, transferrin and alpha1-antitrypsin [40,42,64].

## Conclusion

Highly informative protein profiles for biomarker discovery have been generated by high throughput analysis of both gastric fluid and exfoliated cellular proteomes using small sample volumes and simple sample processing. Identification of pepsinogens A and C among prominent down-regulated markers in gastric cancer fluid samples strengthens the biological plausibility of the proteomic signature we report for gastric cancer diagnosis. A future large cohort study is needed to confirm these results. It will be particularly important to incorporate multiple mucosal biopsies at standard sites and closely follow up patients with clinically benign gastric disorders but whose proteomic profiles are cancer-like. Given the high curative potential of early stage gastric cancer, documented false negative gastroscopic diagnosis and the expense of endoscopic surveillance of all gastric ulcers, a test that provides a pan-gastric molecular biopsy could be a clinically useful supplement to conventional gastroscopy. Proteomic signatures that distinguish benign from malignant disorders, and identify early stage cancer and pre-malignant gastric lesions with high sensitivity and specificity could make a significant contribution to reducing mortality from the second most common cause of global cancer deaths.

## Competing interests

The authors declare that they have no competing interests.

## Authors' contributions

OLK designed the study and wrote the manuscript; TTY analysed all samples, processed data and co-drafted the manuscript; WHC, WWK and HSO collected all samples; SYT reviewed the histopathology and performed *H. pylori *immunostaining; AKHE extracted clinical information; PH performed statistical data analysis; RV assisted in sample processing; MFH, WHN and SYK performed ELISA validation experiments; MPK reviewed historical histopathological records.

## Pre-publication history

The pre-publication history for this paper can be accessed here:



## Supplementary Material

Additional file 1 **Differentially expressed 106 marker peaks in gastric cancer and benign gastric fluids (training set)** Click here for file

Additional file 2 **Full image for Figure**3 Click here for file

Additional file 3 **Differentially expressed 106 marker peaks in gastric cancer and benign gastric fluids (validation set)** Click here for file

Additional file 4 **Full image for Figure**7 Click here for file
