# Supplementary material for: The distinctive gastric fluid proteome in gastric cancer reveals a multi-biomarker diagnostic profile
Source: BMC Med Genomics. 2008 Oct 25;1:54. doi: 10.1186/1755-8794-1-54 (PMC2584050; doi:10.1186/1755-8794-1-54)
Supplement: Additional file 1 — Differentially expressed 106 marker peaks in gastric cancer and benign gastric fluids (training set) [file 1755-8794-1-54-S1.doc]

## Supplementary Table 1

## Differentially expressed 106 marker peaks in gastric cancer and benign gastric fluids (training set)

| *m/z* | Av. peak intensity (SD) | | SAM  score | *m/z* | Av. peak intensity (SD) | | SAM  score |
| --- | --- | --- | --- | --- | --- | --- | --- |
|  | Benign | Cancer |  |  | Benign | Cancer |  |
| 3530 | 0.631  (0.451) | 1.570  (1.454) | 3.383 | 13189 | 1.302  (1.993) | 0.431  (0.425) | 2.842 |
| 3754 | 0.671  (0.493) | 1.572  (1.190) | 3.638 | 6806 | 0.716  (0.472) | 0.255  (0.179) | 4.024 |
| 6863 | 0.099  (0.075) | 0.238  (0.249) | 2.679 | 10431 | 0.140  (0.111) | 0.279  (0.239) | 2.145 |
| 3531 | 0.453  (0.248) | 1.338  (1.286) | 3.754 | 16028 | 0.402  (0.411) | 1.158  (1.325) | 2.909 |
| 3737 | 0.232  (0.118) | 0.543  (0.497) | 3.314 | 2428 | 5.709  (4.233) | 1.189  (1.582) | 4.268 |
| 5722 | 0.195  (0.182) | 0.496  (0.630) | 2.589 | 18343 | 0.969  (1.109) | 0.336  (0.343) | 2.909 |
| 2726 | 3.258  (2.912) | 0.928  (1.236) | 3.176 | 4956 | 0.179  (0.214) | 0.068  (0.028) | 2.062 |
| 13392 | 0.173  (0.186) | 0.460  (0.459) | 3.017 | 4004 | 9.137  (7.856) | 1.742  (3.577) | 3.732 |
| 12673 | 0.250  (0.160) | 0.690  (0.480) | 3.401 | 2214 | 7.338  (7.279) | 1.322  (1.644) | 3.416 |
| 13155 | 0.989  (1.028) | 2.969  (3.643) | 2.781 | 4007 | 2.817  (4.471) | 0.661  (0.943) | 2.009 |
| 1831 | 0.346  (0.284) | 1.213  (1.419) | 3.252 | 2237 | 0.874  (0.521) | 0.480  (0.300) | 2.811 |
| 3412 | 0.912  (0.884) | 1.972  (1.514) | 3.046 | 1884 | 8.826  (6.318) | 2.699  (4.045) | 3.630 |
| 6860 | 0.102  (0.073) | 0.208  (0.133) | 3.252 | 4050 | 3.213  (3.350) | 1.181  (1.742) | 2.357 |
| 2985 | 2.788  (2.955) | 0.610  (0.764) | 3.043 | 2230 | 0.932  (1.101) | 2.784  (2.756) | 2.681 |
| 6824 | 0.177  (0.177) | 0.605  (0.913) | 2.634 | 2096 | 0.912  (0.613) | 0.384  (0.231) | 3.460 |
| 13221 | 0.078  (0.061) | 0.170  (0.142) | 2.760 | 1781 | 0.221  (0.164) | 0.424  (0.279) | 2.850 |
| 6351 | 0.073  (0.030) | 0.193  (0.230) | 2.056 | 4427 | 0.262  (0.190) | 0.874  (1.207) | 2.109 |
| 5566 | 0.340  (4.473) | 0.083  (0.041) | 2.215 | 3927 | 5.485  (6.497) | 0.894  (1.357) | 2.941 |
| 4933 | 3.915  (3.134) | 0.740  (1.226) | 4.042 | 2199 | 2.151  (2.913) | 7.640  (6.312) | 3.425 |
| 3068 | 1.134  (1.732) | 2.591  (1.912) | 2.628 | 2450 | 0.356  (0.215) | 1.578  (1.436) | 3.521 |
| 2936 | 0.716  (0.736) | 4.882  (4.296) | 4.022 | 2634 | 0.248  (0.096) | 0.435  (0.344) | 2.893 |
| 6657 | 1.416  (1.182) | 0.294  (0.371) | 3.850 | 2267 | 2.595  (2.774) | 0.965  (1.242) | 2.315 |
| 3968 | 4.737  (4.049) | 1.258  (2.281) | 3.322 | 2433 | 2.974  (4.260) | 12.596  (8.485) | 4.394 |
| 3605 | 0.502  (0.355) | 1.106  (1.013) | 3.151 | 2436 | 1.708  (1.688) | 0.615  (0.726) | 2.563 |
| 8296 | 0.925  (0.693) | 2.219  (1.162) | 4.626 | 2090 | 0.527  (0.547) | 2.164  (1.704) | 3.867 |
| 6780 | 0.616  (0.272) | 0.992  (0.295) | 4.510 | 2435 | 2.055  (2.800) | 0.684  (0.693) | 2.011 |
| 4850 | 0.667  (0.794) | 0.270  (0.190) | 2.033 | 2191 | 1.198  (1.294) | 0.528  (0.444) | 2.079 |
| 18287 | 0.020  (0.013) | 0.060  (0.049) | 2.964 | 4682 | 0.474  (0.542) | 2.006  (1.258) | 4.749 |
| 12398 | 0.030  (0.015) | 0.066  (0.044) | 2.944 | 2339 | 0.605  (0.630) | 1.678  (1.260) | 3.280 |
| 4359 | 0.488  (0.444) | 0.191  (0.197) | 2.607 | 2394 | 20.700  (13.473) | 5.552  (8.542) | 4.207 |
| 3608 | 1.503  (1.132) | 0.535  (0.470) | 3.419 | 4808 | 1.613  (1.346) | 0.335  (0.422) | 3.850 |
| 5720 | 0.501  (0.959) | 2.238  (2.082) | 3.966 | 2175 | 0.447  (0.414) | 1.303  (0.877) | 3.770 |
| 3372 | 1.478  (1.383) | 3.768  (3.430) | 3.447 | 1885 | 0.813  (0.828) | 2.947  (2.406) | 3.322 |
| 16036 | 0.026  (0.017) | 0.078  (0.057) | 3.383 | 4464 | 1.768  (1.512) | 0.491  (0.542) | 3.389 |
| 5863 | 0.285  (0.182) | 0.640  (0.509) | 3.622 | 2472 | 0.833  (0.567) | 0.309  (0.217) | 3.590 |
| 4504 | 0.737  (0.750) | 0.244  (0.235) | 2.040 | 2352 | 2.011  (1.686) | 0.549  (0.515) | 3.508 |
| 7451 | 0.090  (0.082) | 0.215  (0.193) | 2.742 | 11720 | 3.540  (2.294) | 1.908  (2.150) | 2.404 |
| 10229 | 0.272  (0.173) | 0.611  (0.486) | 3.622 | 2049 | 0.864  (0.903) | 1.651  (1.142) | 2.412 |
| 7565 | 0.179  (0.149) | 0.409  (0.283) | 3.603 | 2354 | 0.161  (0.125) | 1.309  (1.196) | 2.831 |
| 15853 | 1.004  (1.092) | 2.630  (2.227) | 3.584 | 2372 | 1.203  (0.742) | 2.738  (2.227) | 2.800 |
| 13700 | 0.895  (0.723) | 0.270  (0.170) | 3.499 | 2594 | 0.851  (0.654) | 0.339  (0.282) | 3.039 |
| 10434 | 0.468  (0.541) | 1.467  (1.173) | 3.324 | 2453 | 0.985  (0.584) | 0.543  (0.372) | 2.775 |
| 6937 | 1.850  (1.674) | 0.555  (0.490) | 3.186 | 1532 | 0.454  (0.338) | 1.302  (1.286) | 2.769 |
| 2840 | 1.155  (0.871) | 0.427  (0.364) | 3.263 | 2233 | 0.369  (0.288) | 0.771  (0.876) | 2.484 |
| 16130 | 1.241  (1.153) | 0.377  (0.387) | 3.039 | 11855 | 1.562  (1.812) | 0.379  (0.327) | 2.730 |
| 2658 | 0.544  (0.629) | 2.079  (1.686) | 3.639 | 2630 | 1.120  (1.581) | 5.267  (5.077) | 3.330 |
| 6358 | 1.241  (1.153) | 0.377  (0.387) | 3.039 | 5160 | 1.005  (1.093) | 2.632  (2.229) | 3.588 |
| 7448 | 0.287  (0.272) | 0.887  (0.866) | 3.501 | 2176 | 1.969  (2.044) | 5.903  (6.014) | 3.520 |
| 10663 | 1.762  (1.563) | 0.310  (0.504) | 2.947 | 2356 | 0.263  (0.367) | 1.052  (1.229) | 3.520 |
| 3443 | 4.361  (4.110) | 8.767  (5.626) | 2.930 | 1505 | 0.276  (0.114) | 0.575  (0.461) | 3.500 |
| 8581 | 0.666  (0.530) | 0.273  (0.176) | 2.945 | 2109 | 0.747  (0.597) | 0.385  (0.352) | 2.239 |
| 6974 | 0.364  (0.364) | 1.208  (1.049) | 2.883 | 2194 | 0.333  (0.287) | 1.371  (1.590) | 2.730 |
| 7156 | 0.504  (0.178) | 0.770  (0.429) | 3.091 | 4068 | 1.683  (1.684) | 0.550  (0.595) | 2.758 |
