# Supplementary figures and images for: The distinctive gastric fluid proteome in gastric cancer reveals a multi-biomarker diagnostic profile
Source: BMC Med Genomics. 2008 Oct 25;1:54. doi: 10.1186/1755-8794-1-54 (PMC2584050; doi:10.1186/1755-8794-1-54)

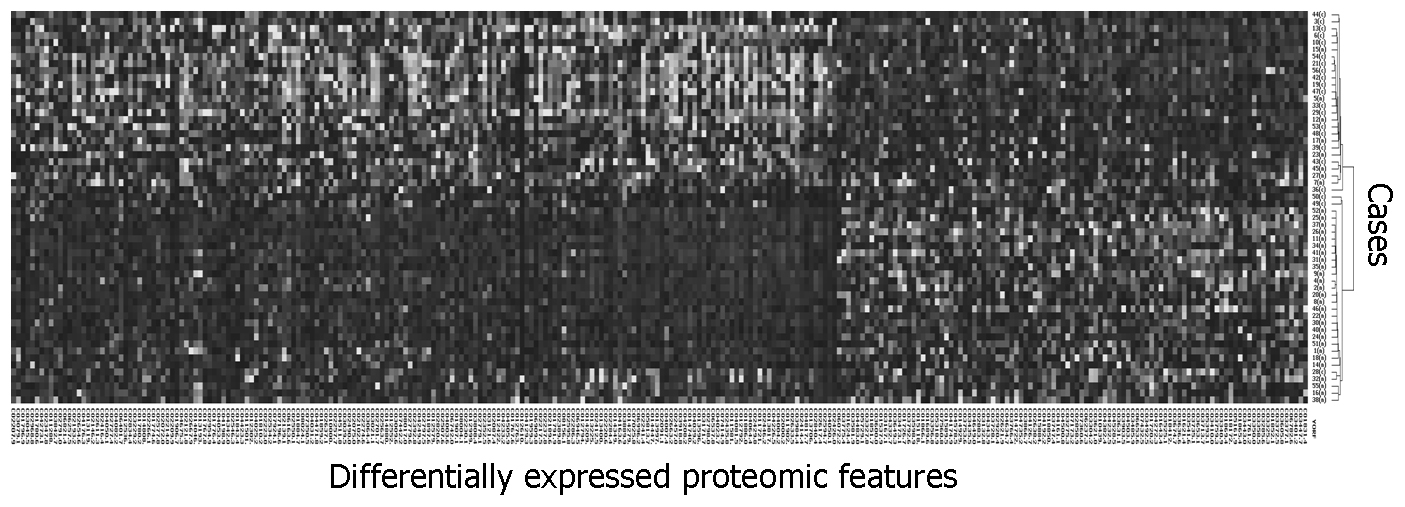

Supplement: Additional file 2 — Full image for Figure3 [file 1755-8794-1-54-S2.jpeg]

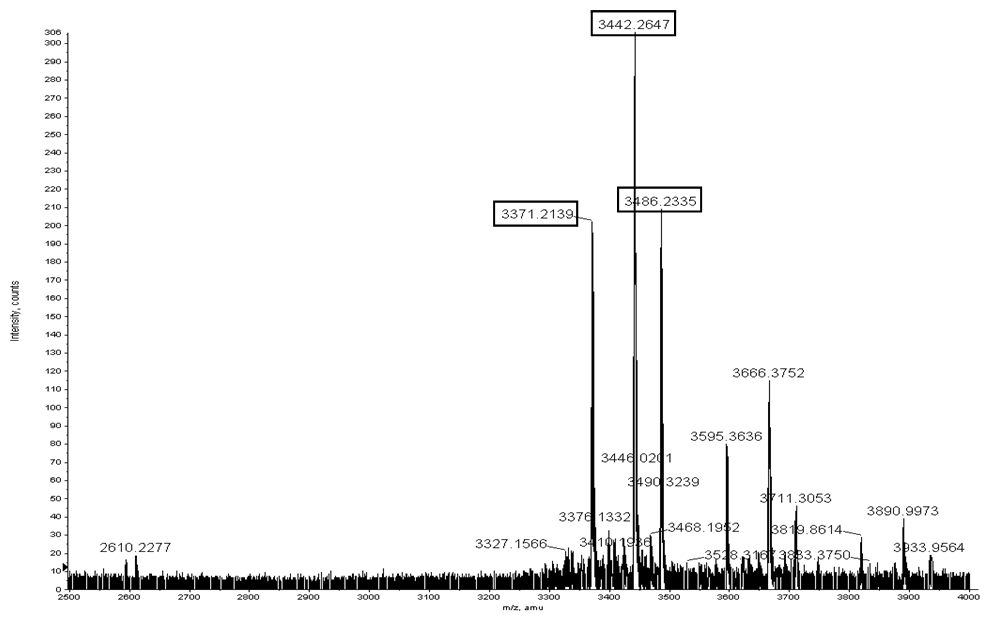

Supplement: Additional file 4 — Full image for Figure7 [file 1755-8794-1-54-S4.jpeg]
