# Supplementary material for: The distinctive gastric fluid proteome in gastric cancer reveals a multi-biomarker diagnostic profile
Source: BMC Med Genomics. 2008 Oct 25;1:54. doi: 10.1186/1755-8794-1-54 (PMC2584050; doi:10.1186/1755-8794-1-54)
Supplement: Additional file 3 — Differentially expressed 106 marker peaks in gastric cancer and benign gastric fluids (validation set) [file 1755-8794-1-54-S3.doc]

## Supplementary Table 2

## Differentially expressed 106 marker peaks in gastric cancer and benign gastric fluids (validation set)

| *m/z* | Av. peak intensity (SD) | | SAM  score | *m/z* | Av. peak intensity (SD) | | SAM  score |
| --- | --- | --- | --- | --- | --- | --- | --- |
|  | Benign | Cancer |  |  | Benign | Cancer |  |
| 3530 | 1.094  (1.254) | 2.468  (2.560) | 2.614 | 13189 | 1.138  (0.845) | 0.559  (0.372) | 2.737 |
| 3754 | 0.568  (0.355) | 1.665  (1.822) | 3.351 | 6806 | 2.637  (2.320) | 0.387  (0.398) | 4.022 |
| 6863 | 0.154  (0.154) | 0.527  (0.795) | 2.634 | 10431 | 1.127  (0.920) | 2.385  (2.287) | 2.168 |
| 3531 | 0.356  (0.223) | 0.907  (0.806) | 3.666 | 16028 | 0.306  (0.253) | 0.852  (1.029) | 2.920 |
| 3737 | 0.884  (0.739) | 2.124  (2.327) | 2.728 | 2428 | 0.827  (0.641) | 0.356  (0.232) | 2.891 |
| 5722 | 0.517  (0.506) | 1.251  (1.621) | 2.517 | 18343 | 1.192  (0.924) | 0.513  (0.334) | 2.890 |
| 2726 | 1.297  (1.239) | 0.437  (0.433) | 2.797 | 4956 | 0.765  (0.731) | 0.454  (0.275) | 2.086 |
| 13392 | 0.330  (0.337) | 0.951  (1.088) | 2.909 | 4004 | 0.309  (0.263) | 0.157  (0.152) | 2.112 |
| 12673 | 10.982  (9.747) | 22.350  (15.047) | 3.160 | 2214 | 0.990  (0.978) | 0.379  (0.293) | 2.528 |
| 13155 | 0.072  (0.056) | 0.162  (0.135) | 2.761 | 4007 | 1.591  (2.004) | 0.482  (0.674) | 2.010 |
| 1831 | 0.380  (0.375) | 1.802  (2.759) | 2.942 | 2237 | 0.927  (0.572) | 0.518  (0.622) | 2.252 |
| 3412 | 1.001  (1.030) | 2.070  (2.095) | 2.480 | 1884 | 3.147  (2.499) | 1.015  (1.167) | 3.356 |
| 6860 | 0.631  (0.223) | 0.964  (0.537) | 3.091 | 4050 | 2.021  (1.369) | 0.512  (0.639) | 4.279 |
| 2985 | 3.508  (3.084) | 1.574  (2.147) | 2.312 | 2230 | 1.339  (1.312) | 2.540  (1.676) | 2.548 |
| 6824 | 2.143  (1.730) | 3.872  (2.337) | 2.630 | 2096 | 1.029  (1.201) | 0.423  (0.352) | 2.056 |
| 13221 | 0.559  (0.372) | 1.137  (0.844) | 2.680 | 1781 | 0.298  (0.404) | 0.955  (0.846) | 3.859 |
| 6351 | 0.336  (0.280) | 0.818  (0.955) | 2.108 | 4427 | 0.446  (0.321) | 0.757  (0.519) | 2.153 |
| 5566 | 0.147  (0.134) | 0.073  (0.036) | 2.088 | 3927 | 11.867  (12.151) | 1.896  (3.613) | 3.374 |
| 4933 | 3.329  (2.665) | 0.629  (1.042) | 4.044 | 2199 | 9.599  (14.475) | 29.742  (19.210) | 3.783 |
| 3068 | 0.770  (1.259) | 2.043  (1.882) | 2.518 | 2450 | 0.767  (0.860) | 4.437  (4.206) | 3.607 |
| 2936 | 0.313  (0.702) | 0.971  (0.640) | 4.024 | 2634 | 0.618  (0.798) | 1.602  (1.935) | 2.668 |
| 6657 | 0.562  (0.469) | 0.117  (0.147) | 3.750 | 2267 | 0.539  (0.443) | 0.296  (0.124) | 2.136 |
| 3968 | 5.083  (5.861) | 1.509  (1.780) | 2.562 | 2433 | 0.435  (0.453) | 1.644  (1.486) | 3.273 |
| 3605 | 0.836  (0.733) | 1.523  (0.814) | 3.007 | 2436 | 4.100  (3.867) | 1.286  (2.000) | 2.832 |
| 8296 | 4.274  (2.681) | 1.011  (1.157) | 4.662 | 2090 | 0.316  (0.250) | 1.002  (0.733) | 3.542 |
| 6780 | 0.712  (0.533) | 1.708  (0.894) | 4.630 | 2435 | 0.899  (0.904) | 0.276  (0.281) | 2.010 |
| 4850 | 0.333  (0.235) | 0.200  (0.153) | 2.011 | 2191 | 1.109  (1.057) | 0.344  (0.277) | 2.962 |
| 18287 | 0.032  (0.012) | 0.077  (0.050) | 2.853 | 4682 | 0.426  (0.487) | 1.802  (1.130) | 4.584 |
| 12398 | 0.051  (0.020) | 0.096  (0.052) | 2.910 | 2339 | 0.701  (0.715) | 2.214  (1.810) | 3.325 |
| 4359 | 0.234  (0.241) | 0.091  (0.032) | 2.361 | 2394 | 5.367  (4.627) | 1.447  (2.228) | 3.321 |
| 3608 | 1.503  (1.297) | 0.455  (0.220) | 3.321 | 4808 | 8.248  (7.092) | 1.573  (3.230) | 3.713 |
| 5720 | 0.512  (0.981) | 2.089  (1.943) | 3.960 | 2175 | 0.631  (1.296) | 3.309  (2.845) | 3.721 |
| 3372 | 3.244  (2.782) | 6.402  (4.049) | 3.184 | 1885 | 2.699  (4.045) | 8.826  (6.318) | 3.630 |
| 16036 | 0.194  (0.182) | 0.495  (0.451) | 3.402 | 4464 | 1.626  (1.471) | 0.488  (0.431) | 3.286 |
| 5863 | 0.167  (0.106) | 0.375  (0.298) | 3.531 | 2472 | 2.257  (2.017) | 0.643  (0.856) | 3.178 |
| 4504 | 0.667 (0.794) | 0.270  (0.190) | 2.033 | 2352 | 0.857  (0.580) | 0.254  (0.118) | 4.262 |
| 7451 | 0.118  (0.081) | 0.385  (0.547) | 2.681 | 11720 | 1.220  (1.281) | 0.420  (0.311) |  |
| 10229 | 0.480  (0.670) | 1.920  (2.259) | 3.603 | 2049 | 1.946  (2.143) | 3.475  (2.638) | 2.049 |
| 7565 | 0.105  (0.062) | 0.236  (0.214) | 3.130 | 2354 | 0.762  (0.762) | 2.092  (1.816) | 2.878 |
| 15853 | 2.434  (2.087) | 4.803  (3.038) | 3.141 | 2372 | 0.516  (0.344) | 1.050  (0.779) | 2.680 |
| 13700 | 2.320  (2.099) | 0.696  (0.615) | 3.186 | 2594 | 3.205  (2.710) | 0.810  (0.999) | 3.541 |
| 10434 | 0.405  (0.422) | 1.531  (1.384) | 3.263 | 2453 | 0.895  (0.855) | 0.302  (0.299) | 2.802 |
| 6937 | 0.998  (0.892) | 0.285  (0.332) | 3.150 | 1532 | 0.953  (0.709) | 2.735  (2.701) | 2.760 |
| 2840 | 0.737  (0.464) | 0.370  (0.245) | 2.947 | 2233 | 0.424  (0.230) | 0.905  (0.811) | 2.517 |
| 16130 | 0.848  (0.630) | 0.417  (0.278) | 2.680 | 11855 | 1.106  (1.283) | 0.268  (0.231) | 2.730 |
| 2658 | 0.759  (0.720) | 3.083  (3.840) | 2.515 | 2630 | 1.034  (1.250) | 2.220  (1.508) | 2.760 |
| 6358 | 1.204  (0.933) | 0.518  (0.338) | 2.880 | 5160 | 0.576  (0.361) | 1.468  (1.305) | 3.566 |
| 7448 | 0.348  (0.301) | 0.863  (0.852) | 3.438 | 2176 | 0.438  (0.181) | 0.912  (0.731) | 3.501 |
| 10663 | 2.342  (2.176) | 0.712  (0.731) | 3.039 | 2356 | 8.015  (7.114) | 16.312  (10.982) | 3.250 |
| 3443 | 4.284  (4.034) | 8.113  (5.015) | 2.894 | 1505 | 0.252  (0.210) | 0.576  (0.399) | 3.601 |
| 8581 | 1.678  (1.489) | 0.528  (0.858) | 2.941 | 2109 | 0.807  (0.598) | 0.434  (0.361) | 2.347 |
| 6974 | 0.087  (0.087) | 0.240  (0.208) | 2.886 | 2194 | 0.392  (0.211) | 0.796  (0.684) | 2.388 |
| 7156 | 0.696  (0.610) | 1.268  (0.678) | 3.004 | 4068 | 1.306  (0.850) | 0.459  (0.367) | 3.944 |
